# Impact of Sleep Apnea Treatment in Patients with Unexplained Syncope: The SINCOSAS Study

**DOI:** 10.3390/jcm15041318

**Published:** 2026-02-07

**Authors:** María-José Muñoz-Martínez, Manuel Casal-Guisande, Bernardo Sopeña, María Torres-Durán, Enrique García-Campo, Dolores Corbacho-Abelaira, Ana Souto-Alonso, Alberto Fernández-Villar

**Affiliations:** 1 Pulmonary Department, Hospital Álvaro Cunqueiro, 36312 Vigo, Spain; maria.luisa.torres.duran@sergas.es (M.T.-D.); alberto.fernandez.villar@sergas.es (A.F.-V.); 2 NeumoVigo I+i Research Group, Galicia Sur Health Research Institute (IIS Galicia Sur), SERGAS-UVIGO, 36312 Vigo, Spain; dcorbacho@povisa.es; 3 Faculty of Medicine and Dentistry, University of Santiago de Compostela, 15782 Santiago de Compostela, Spain; bernardo.sopena.perez-arguelles@sergas.es; 4 Centro de Investigación Biomédica en Red, CIBERES ISCIII, 28029 Madrid, Spain; 5 Department of Design in Engineering, University of Vigo, 36208 Vigo, Spain; 6 Internal Medicine Department, Hospital Clínico Universitario de Santiago de Compostela, 15782 Santiago de Compostela, Spain; 7 Cardiology Department, Hospital Álvaro Cunqueiro, 36312 Vigo, Spain; enrique.garcia.campo@sergas.es; 8 Pulmonary Department, Hospital Ribera Povisa, 36211 Vigo, Spain; 9 Pulmonary Department, Hospital Universitario de A Coruña, 15006 A Coruña, Spain; ana.souto.alonso@sergas.es; 10 School of Industrial Engineering, University of Vigo, 36310 Vigo, Spain

**Keywords:** sleep apnea, syncope, heart rate variability, autonomic nervous system

## Abstract

**Objectives:** Unexplained syncope (US) persists despite extensive diagnostic evaluations, with autonomic dysfunction as a central mechanism. Sleep apnea (SA) may contribute through intermittent hypoxemia and autonomic imbalance. We evaluated the impact of SA treatment on syncope recurrence, nocturnal heart rate variability (HRV), and quality of life in patients with US. **Methods**: We conducted a prospective multicentre study in three hospitals in Galicia (Spain), including adults with US who underwent home respiratory polygraphy. SA was diagnosed according to guideline criteria, and treatment was prescribed when indicated (positive airway pressure therapy, positional therapy, and/or weight management). Symptoms, syncope burden, nocturnal heart rate variability derived from the ECG signal, and quality of life (SF-36 and a 0–100 visual analogue scale) were assessed at baseline and after 12 months. **Results**: Of 141 patients, 99 met treatment criteria, and 67 completed the 12-month follow-up. Mean age was 64.5 years; 59.6% were men; mean AHI was 25.9/h. After therapy, daytime sleepiness (Epworth score decreased from 8 to 5; *p* = 0.001), fatigue, nocturnal awakenings, and syncopal episodes decreased from 62.6% to 16.2%, 56.6% to 16.2%, and 3 to 0, respectively (all *p* < 0.001). HRV showed increased RR interval (*p* < 0.001) and RMSSD (*p* = 0.04). Quality of life improved in vitality (SF-36 vitality domain increased from 44 to 50; *p* = 0.02) and on the visual analogue scale (0–100: 50 to 70; *p* = 0.002). **Conclusions**: In this prospective cohort of patients with US and SA, therapy for SA was associated with fewer syncope recurrences, improvements in nocturnal respiratory indices, and selected heart rate variability measures, and better self-reported fatigue and vitality. Given the single-arm design and potential adherence and selection biases, these findings should be interpreted with caution and warrant confirmation in controlled studies.

## 1. Introduction

Syncope is a common reason for medical consultation and is defined as a transient loss of consciousness due to global cerebral hypoperfusion, with abrupt onset, short duration, and complete spontaneous recovery [1]. Among the mechanisms involved, dysfunction of the autonomic nervous system (ANS) plays a key role. However, in a considerable number of patients, even after an extensive diagnostic evaluation, the underlying cause cannot be identified. These cases, classified as unexplained syncope (US), represent a significant clinical challenge and are associated with impaired quality of life, as well as challenges in establishing effective strategies to prevent recurrences [2].

Sleep apnea (SA) is a chronic respiratory disorder affecting up to 30% of the adult population in developed countries [3]. It is characterised by recurrent episodes of upper airway collapse during sleep, leading to intermittent hypoxemia, sleep fragmentation, and fluctuations in intrathoracic pressure and heart rate [4]. These alterations result in persistent sympathetic activation and have been linked to an increased risk of arterial hypertension [5], arrhythmias [6], bradycardia [7], ischemic heart disease [8,9,10], and endothelial dysfunction [11]. Moreover, the sympathetic–parasympathetic imbalance induced by SA may contribute to the occurrence of vasovagal or bradycardic syncope [6,12]. SA is also associated with impaired quality of life, an effect that may be reversed with appropriate therapy [13,14,15].

Recent studies have shown a high prevalence of SA in patients with US, suggesting a clinical link between the two conditions [16]. Mechanisms such as intermittent hypoxemia, sleep fragmentation, and repeated variations in intrathoracic pressure have been proposed to induce chronic autonomic dysfunction [17], thereby increasing susceptibility to syncope in predisposed individuals. In this context, heart rate variability (HRV) represents a useful non-invasive biomarker to explore autonomic regulation during sleep. Preliminary observations and case series suggest that continuous positive airway pressure (CPAP) therapy may modulate autonomic tone and be associated with reduced recurrence of syncope or vagal events during wakefulness [18,19]. Although some reports have suggested that SA treatment may improve syncope recurrence and autonomic modulation, no prospective studies have systematically evaluated this in patients with US to date. In this context, our objective was to analyse, in a cohort of patients with US and SA, the impact of SA therapy on nocturnal HRV, syncope recurrence, and quality of life after 12 months of follow-up. We hypothesised that correction of SA may enhance nocturnal autonomic stability, reduce the risk of recurrent syncope, and improve functional status, thereby supporting the integration of sleep assessment into the multidisciplinary management of US.

## 2. Materials and Methods

### 2.1. Study Design and Population

We conducted a prospective, longitudinal, multicentre study in three hospitals in Galicia (Spain): Álvaro Cunqueiro University Hospital (Vigo), Ribera Povisa Hospital (Vigo), and A Coruña University Hospital Complex. Patients were enrolled between June 2019 and May 2024, all within the framework of the SINCOSAS Project [16,20,21]. A total of 133 patients were recruited at Álvaro Cunqueiro University Hospital, 4 patients at Ribera Povisa Hospital, and 4 patients at A Coruña University Hospital Complex.

Consecutive adult patients (≥18 years) with US after a comprehensive evaluation according to current guidelines were recruited, including clinical history and physical examination, 12-lead ECG, and additional testing as clinically indicated (e.g., echocardiography, ambulatory ECG monitoring, and neurological assessment) [1]. Some participants were still awaiting prolonged rhythm monitoring (e.g., implantable loop recorder) at the time of recruitment. Patients were referred from cardiology outpatient clinics (including those on the waiting list for implantable loop recorder placement), neurology, pulmonology, and emergency departments. Subjects with a previous diagnosis of epilepsy or active use of psychoactive substances were excluded.

**Figure 1:**
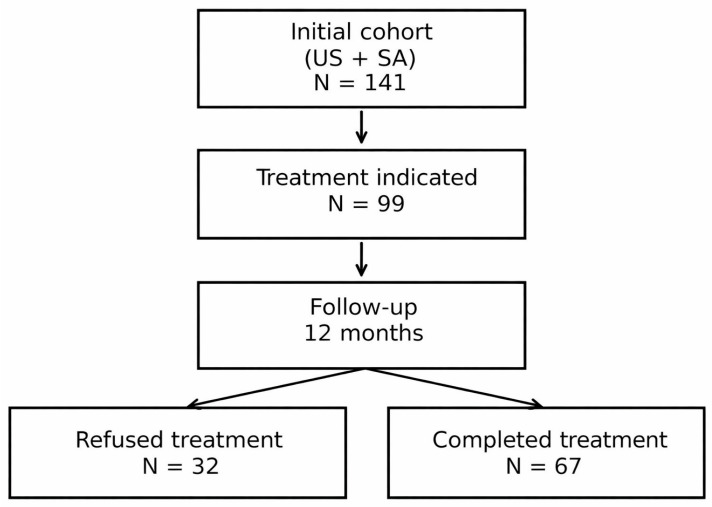
Flowchart of patient selection and treatment outcomes.

For the analysis, three patient groups were considered: the global cohort, including all subjects with US and SA assessed at baseline; the treatment-indicated cohort, comprising those meeting criteria for specific SA therapy according to clinical guidelines [4]; and the follow-up cohort, consisting of treated patients who completed both baseline and 12-month assessments (see Figure 1), on whom paired comparisons were performed.

Quality of life was assessed using a visual analog scale (0–100) [22] and the SF-36 questionnaire [23], which comprises eight domains (physical functioning, role-physical, bodily pain, general health, vitality, social functioning, role-emotional, and mental health). For analysis, these domains were grouped into two components: physical (physical functioning, role-physical, bodily pain, general health) and mental (vitality, social functioning, role-emotional, mental health).

The study was approved by the Research Ethics Committee of Galicia (2019/048), and all participants provided written informed consent in accordance with the Declaration of Helsinki.

### 2.2. Clinical Data Collection

Demographic and anthropometric variables were recorded, including age, sex, and body mass index (BMI). Relevant medical history was documented: arterial hypertension, ischemic or valvular heart disease, atrial fibrillation, stroke, diabetes mellitus, dyslipidaemia, chronic obstructive pulmonary disease (COPD), and asthma. Smoking status was assessed based on current habit and pack-years.

Symptoms suggestive of SA were collected, including excessive daytime sleepiness measured by the Epworth Sleepiness Scale, nocturnal awakenings, non-restorative sleep, and daytime fatigue.

Syncope burden was determined from the total number of episodes occurring in the 12 months prior to the baseline visit. During follow-up, the number of syncopal episodes occurring in the 12 months after inclusion was recorded. Participants continued standard-of-care evaluation and management for syncope at the discretion of the treating physicians. Any new diagnostic findings leading to reclassification of syncope aetiology were recorded when available.

Traumatic events were defined as syncopal episodes that resulted in an injury requiring medical evaluation in either the hospital emergency department or a primary care urgent care facility.

### 2.3. Home Respiratory Polygraphy, Diagnosis, and Treatment of Sleep Apnea

All participants underwent home respiratory polygraphy with the Embletta^®^ MPR system (Natus Medical Inc., Middleton, WI, USA), which includes synchronised electrocardiogram (ECG) recording and automatic analysis of heart rate variability. Recordings covered the period from 00:00 to 07:00 h and were manually reviewed to ensure signal quality and correct classification of respiratory events.

The following indices were analysed: apnea-hypopnea index (AHI), time with oxygen saturation <90% (T90), desaturation index ≥3% (ODI3), and the number of events by type (obstructive, central, mixed apneas, and hypopneas).

The diagnosis of SA was established according to the SEPAR 2021 consensus criteria [4]: AHI ≥ 15 events/hour with predominance of obstructive events, or AHI ≥ 5 events/hour in the presence of compatible clinical symptoms. Severity was classified as mild (AHI 5–14.9/h), moderate (15–29.9/h), or severe (≥30/h). Positional obstructive sleep apnea was defined as a supine AHI at least twice the non-supine AHI.

Therapeutic indication followed SEPAR guideline recommendations [4]. Positive airway pressure therapy (CPAP, BiPAP, or ASV) was indicated in patients with an AHI ≥ 15 events/hour, or AHI ≥ 5 events/hour in the presence of compatible symptoms or cardiovascular comorbidities. Positional therapy was prescribed in patients with positional obstructive sleep apnea, particularly in mild to moderate disease. Weight management was recommended as an adjunctive strategy in overweight or obese patients. Among patients treated with pressure devices, only those with use of >4 h per day were included in the analysis.

### 2.4. Heart Rate Variability Analysis

Nocturnal HRV was analysed from the continuous ECG recording included in the respiratory polygraphy (Embletta^®^ MPR). Five-minute segments were selected from the total 7-hour recording period (00:00–07:00).

Patients with atrial fibrillation were not excluded; therefore, HRV results in this subgroup should be interpreted cautiously.

Time-domain parameters were calculated, including mean RR intervals, standard deviation of all normal intervals (SDNN), standard deviation of 5-min mean NN intervals (SDANN), root mean square of successive differences (RMSSD), SDNN index, number of intervals differing by more than 50 ms from the previous one (NN50), the percentage of NN50 (pNN50), and the triangular index of HRV.

In the frequency domain, total power and very low frequency (VLF), low frequency (LF), and high frequency (HF) bands were evaluated. Interpretation of the results followed the recommendations of the European Society of Cardiology and the European Heart Rhythm Association [1].

Exploratorily, reduced HRV was considered when decreases were observed in time-domain parameters and HF (parasympathetic activity), together with increases in VLF and LF (sympathetic activity).

### 2.5. Statistical Analysis

Normality of quantitative variables was assessed using the Shapiro–Wilk test. Since respiratory polygraphy parameters did not follow a normal distribution, paired comparisons of quantitative variables before and after treatment were performed with the non-parametric Wilcoxon signed-rank test.

Paired categorical variables were analysed using McNemar’s test. A two-tailed *p* value < 0.05 was considered statistically significant. Effect sizes were also calculated and interpreted according to Cohen’s criteria.

Quantitative variables were described as mean and standard deviation when normally distributed, and as median and interquartile range (Q1–Q3) otherwise. Qualitative variables were expressed as absolute frequencies and percentages.

Baseline characteristics are presented as mean ± SD for descriptive purposes, whereas pre–post comparisons in the follow-up cohort were analysed using paired non-parametric tests and are presented as median (Q1–Q3).

All analyses were performed using SPSS version 25.0 (IBM Corp., Armonk, NY, USA).

## 3. Results

### 3.1. Study Population and Treatment Exposure

A total of 141 patients with US and SA were included. Of these, 99 met treatment criteria according to clinical guidelines: 2 received weight management intervention, 3 positional therapy, and 94 positive airway pressure (77 CPAP, 11 auto-CPAP, 5 servo-ventilator, and 1 BiPAP). Among the 99 patients with therapeutic indication, 32 (32.3%) refused treatment due to intolerance to pressure devices and 67 completed 12-month follow-up.

### 3.2. Baseline Characteristics

Of the 141 patients included, baseline characteristics are presented for the 99 with treatment indication. Mean age was 64.5 years, and 59.6% were male. Mean BMI was 29.8 kg/m^2^. Regarding smoking status, 46.5% were non-smokers, 37.4% ex-smokers, and 16.3% active smokers, with a mean of 35.9 pack-years among smokers and ex-smokers. The most frequent comorbidities were arterial hypertension (48.5%), dyslipidaemia (49.5%), atrial fibrillation (20.2%), and diabetes mellitus (17.1%).

At baseline clinical evaluation, the Epworth Sleepiness Scale showed a mean of 9.24 points; 62.6% reported daytime fatigue, 56.6% nocturnal awakenings, and 58.6% non-restorative sleep. The mean number of reported syncopal episodes was 8.86.

In respiratory polygraphy, mean AHI was 25.9/h, ODI3 24.8, and T90 15.1%. Nocturnal HRV values included a mean RR interval of 928.5 ms, SDNN 127.2 ms, RMSSD 140.1 ms, and total power 25,674.4 ms^2^.

Complete clinical and polygraphy characteristics are shown in Table 1 and Table 2.

### 3.3. Clinical Outcomes at 12 Months

Clinical, respiratory, autonomic, and quality-of-life outcomes at 12 months in the follow-up subcohort (n = 67) are summarised in Table 3. The main findings are described below.

#### 3.3.1. Clinical Outcomes

At 12-month follow-up, significant symptom improvement was observed. The mean Epworth score decreased from 8 to 5 (*p* = 0.001), daytime fatigue declined from 62.6% to 16.2% (*p* < 0.001), nocturnal awakenings decreased from 56.6% to 16.2% (*p* < 0.001), and lack of concentration declined from 43.4% to 9.1% (*p* < 0.001). Syncope burden fell from a median of 3–0 episodes (*p* < 0.001). Effect sizes (r) for the Wilcoxon signed-rank test were calculated for the main outcomes of interest; the reduction in syncope burden showed a very large effect size (r = 0.85), indicating a clinically meaningful improvement.

#### 3.3.2. Respiratory Parameters

Respiratory parameters showed significant reductions: Mean AHI decreased from 23.5 to 5.0/h (*p* < 0.001), ODI3 from 22.9 to 5.2 (*p* < 0.001), and T90 from 6.5% to 0.2% (*p* < 0.001). Significant decreases were also observed in obstructive, central, and mixed apneas, as well as in hypopneas (all *p* ≤ 0.04).

#### 3.3.3. Heart Rate Variability

In HRV metrics, mean RR interval increased (*p* < 0.001) and RMSSD improved (*p* = 0.04). No significant changes were found in SDNN, SDANN, pNN50, total power, VLF, LF, HF, or LF/HF ratio. Effect sizes (r) for the Wilcoxon signed-rank test indicated a large effect size for changes in the mean RR interval (r = 0.54) and a small effect size for changes in RMSSD (r = 0.19).

#### 3.3.4. Quality of Life

The mean visual analogue scale of quality of life showed significant improvement, increasing from 50 to 70; *p* = 0.002). In the SF-36, only the vitality domain showed a significant increase, from 44 to 50 (*p* = 0.02), while no relevant changes were observed in the other domains.

## 4. Discussion

### 4.1. Main Findings

In this cohort of patients with US and SA, SA therapy was associated with (i) a significant reduction in syncope recurrence, (ii) objective improvement in nocturnal respiratory parameters (AHI, ODI3, and T90), (iii) an increase in mean RR interval and RMSSD in HRV, and (iv) improved quality of life, mainly in vitality and on the visual analogue scale. Taken together, these findings may suggest changes in nocturnal autonomic modulation after therapy; however, the clinical significance of the observed changes in RMSSD and nocturnal heart rate remains uncertain. Effect size analysis added clinically relevant information beyond statistical significance. SA therapy was associated with a very large effect size for syncope reduction (r = 0.85), indicating a clinical impact. In contrast, autonomic changes were more modest: the mean RR interval showed a large effect size (r = 0.54), whereas RMSSD exhibited only a small effect size (r = 0.19). This pattern suggests that the marked clinical improvement in syncope recurrence was accompanied by subtler and more variable changes in nocturnal autonomic modulation, supporting the interpretation of HRV findings as complementary and hypothesis-generating rather than definitive mechanistic evidence.

Quality-of-life assessment was included as a secondary, patient-centred outcome to complement clinical and physiological findings. US is known to impair daily functioning, vitality, and perceived health status, even in the absence of frequent recurrences, and SA has an independent negative impact on quality of life. In this context, the observed improvement in vitality and in the visual analogue scale likely reflects the combined effect of reduced syncope burden, improved nocturnal breathing, and decreased sleep fragmentation. The absence of significant changes in most SF-36 domains further supports a cautious interpretation, reinforcing that quality-of-life results should be viewed as supportive indicators of clinical benefit rather than as primary efficacy outcomes.

### 4.2. Relation to the Literature

This work is part of the SINCOSAS project [16,20,21], which previously showed a high prevalence of SA in patients with US. Unlike that earlier phase, focused on prevalence, here we analysed longitudinal outcomes after therapy. In line with Gula et al. [24], who observed reductions in LF and HF in moderate SA compatible with “exhaustion” of autonomic control, our results suggest that correction of SA may be accompanied by signs of vagal tone recovery (increase in RMSSD), although without global changes in frequency bands. Vagal rebound phenomena linked to sleep architecture [25] are also plausible. The meta-analysis by Guo et al. [26] demonstrated that CPAP reduces sympathetic activation (decrease in LF, decrease in LF/HF) and enhances parasympathetic activity (increase in HF), even beyond immediate device use. These findings support an autonomic effect that extends beyond nocturnal application and reinforce the notion of a sustained vagal benefit, although the magnitude and persistence of this effect outside the nocturnal context remain debated. In line with this, our increase in RMSSD—a time-domain metric related to vagal modulation—supports the hypothesis of partial normalization of autonomic control in treated patients, although we did not observe significant changes in HF, LF, or LF/HF. [27].

Regarding quality of life, previous studies reported improvement with CPAP [13,15] and also in patients treated for syncope [2]. In our cohort, the improvement was most evident in vitality and in the visual analogue scale, which may reflect the impact of reduced hypoxemia/awakenings and lower syncope burden.

Finally, clinical reports and small case series have suggested that treating SA may reduce syncope recurrence in US [18,28]. Our prospective data point in the same direction, although causality cannot be inferred due to the absence of a control group.

### 4.3. Pathophysiological Interpretation

Intermittent hypoxemia, sleep fragmentation, and intrathoracic pressure swings typical of SA may induce chronic autonomic dysfunction [17]. HRV provides a non-invasive biomarker of autonomic control: the increase in RMSSD and RR interval after therapy suggests enhanced vagal modulation and reduced nocturnal sympathetic load, which could decrease susceptibility to vasovagal or bradycardic responses and, consequently, syncope recurrence. These findings are consistent with the circadian rhythm of HRV [29] and with the feasibility of assessing nocturnal autonomic modulation using the nighttime segment of 24-hour Holter monitoring, routinely employed in the management of US [30]. Various sleep disorders, including SA [29], restless legs syndrome [31], insomnia [32], and others [33], may disrupt this pattern, underscoring the need to systematically consider sleep in the approach to US.

### 4.4. Clinical Implications

Our results support the integration of SA assessment in patients with US, particularly when suggestive symptoms are present. The improvement in syncope and vitality after therapy, together with signals of nocturnal autonomic rebalancing, point towards a potential clinical benefit. In practice, home respiratory polygraphy enables accessible screening, and HRV derived from nocturnal ECG provides complementary pathophysiological information that may have prognostic and follow-up value. This approach could help reshape clinical practice in patients with US, systematically incorporating sleep assessment into diagnostic and management algorithms and offering therapeutic opportunities not previously considered.

### 4.5. Strengths and Limitations

The main strengths of this study include its multicentre design and multidisciplinary approach, integrating the participation of specialists in cardiology, pulmonology, and sleep medicine. Validated tools were used for the diagnosis of SA, assessment of HRV, and measurement of quality of life, reinforcing the robustness of the findings. Another relevant strength is the simultaneous evaluation of clinical, respiratory, autonomic, and quality-of-life outcomes within a single follow-up protocol, providing a comprehensive view of the impact of SA therapy in patients with US.

Nevertheless, several limitations should be considered when interpreting the results. First, the absence of an untreated control group precludes attributing to the observed changes exclusively to SA therapy. Second, the treatment discontinuation rate was high (32%), which may have introduced selection bias in the longitudinal analysis. Moreover, although trends towards improvement were observed in several HRV parameters, the relatively limited sample size may have reduced the ability to detect statistically significant changes in frequency-domain measures. In addition, the use of home respiratory polygraphy instead of polysomnography (the reference standard) may have introduced misclassification of disease severity. Another limitation is the lack of universally accepted reference values for nocturnal HRV, which hampers comparative interpretation of the results. Finally, although the study was multicentre, most patients were recruited from a single hospital, which may limit the external validity of the findings to other clinical settings.

### 4.6. Future Directions

Randomised controlled trials are needed to evaluate the effect of SA therapy on syncope recurrence and autonomic biomarkers. Stratification by adherence, baseline severity of SA, clinical phenotypes, and comorbidities (e.g., atrial fibrillation) would be useful. The role of HRV as a marker of response and prognosis requires further validation, ideally with standardised nocturnal recordings and comparison with polysomnography. It would also be relevant to compare different diagnostic strategies—24-h Holter, polygraphy, or polysomnography—to determine which provides the best performance in nocturnal autonomic assessment and its prognostic value in patients with US.

## 5. Conclusions

The SINCOSAS study suggests that SA therapy in patients with US was associated with fewer recurrent syncopal episodes, improvements in respiratory indices, and changes in selected nocturnal HRV parameters, along with better patient-reported symptoms and vitality. These findings should be interpreted with caution and considered hypothesis-generating. Controlled studies are needed to confirm causality and to identify which patient subgroups may benefit most.

## Figures and Tables

**Table 1:** Baseline demographic and clinical characteristics of the study population.

Variable	Baseline n = 99 ** (%)
Sex, male	58 (59.8%)
Age (years)	67.0 (56.0–75.0)
BMI (kg/m^2^)	29.2 (26.2–32.4)
Never smoker	49 (50.5%)
Ex-smoker	14 (14.4%)
Current smoker	34 (35.1%)
Pack-years (smokers and ex-smokers)	30.0 (17.0–54.0)
Ischaemic heart disease	14 (14.4%)
Valvular heart disease	1 (1.0%)
Atrial fibrillation	21 (21.6%)
High blood pressure	49 (50.5%)
Stroke	1 (1.0%)
Diabetes mellitus	13 (13.4%)
Dyslipidaemia	47 (48.5%)
COPD	5 (5.2%)
Asthma	7 (7.2%)
Epworth Sleepiness Scale	8.0 (3.0–13.0)
Daytime fatigue	60 (61.9%)
Nocturnal awakenings	50 (51.5%)
Lack of concentration	44 (45.4%)
Observed apneas	27 (27.8%)
Nocturnal choking episodes	15 (15.5%)
Non-restorative sleep	55 (56.7%)

Abbreviations: COPD—chronic obstructive pulmonary disease; BMI—body mass index; ** “n” denotes the number of patients reporting each variable.

**Table 2:** Baseline respiratory polygraphy and heart rate variability parameters of the study population.

Variable	Baseline n = 99 ** Median (Q1–Q3)
Total number of syncopes	5 (3–10)
Traumatic events	1 (0–1)
AHI (events/hour)	21.6 (9.1–35.3)
ODI3	21 (8.9–35.5)
T90 (%)	4.9 (0.7–13.5)
N obstructive apneas	9 (2.0–56)
N central apneas	1 (0–6)
N mixed apneas	0 (0–2)
N hypopneas	81 (46–145)
Mean RR (ms)	941 (821–1016)
SDNN (ms)	99.5 (73.2–160.5)
SDNN index (ms)	72.5 (49.5–137.0)
RMSSD (ms)	70.5 (42.5–175.8)
NN50	2556.0 (561.2–8097.2)
%NN50	11.8 (3–37.2)
SDANN (ms)	56.5 (40–108)
Average total power (ms^2^)	20,520 (12,906.0–33,851)
Average VLF power (ms^2^)	8581 (2619–17,503.5)
Average LF power (ms^2^)	7126 (3466.0–10,768)
Average HF power (ms^2^)	3304 (2199–5221)
LF/HF	1.9 (1.1–3.3)
Triangular index HRV	16 (11–20)
Visual analogue scale (0–100)	50 (40–70)
SF-36 physical functioning	60 (35–80)
SF-36 role physical	50 (31–75)
SF-36 bodily pain	51 (31–68.5)
SF-36 general health	45 (30–55)
SF-36 vitality	38 (19–56)
SF-36 social functioning	63 (38–97)
SF-36 role emotional	75 (50–100)
SF-36 mental health	55 (50–70)

Abbreviations: AHI: apnea-hypopnea index; ODI3—desaturation index >3%; T90—desaturation time below 90%; ms—milliseconds; HRV—heart rate variability; RR—RR intervals; SDNN—standard deviation of RRs; SDANN—standard deviation of the averages of RR intervals across all segments per minute of the entire analysis interval; RMSSD—square root of the mean value of the sum of the squared differences of all successive RR intervals; NN50—number of consecutive intervals varying by more than 50 ms; HF—high frequency range power; VLF—very low-frequency range power; LF—low-frequency range power. ** “n” denotes the number of patients reporting each variable.

**Table 3:** Differences before and after 12 months (n = 67).

	Baseline Median (Q1–Q3)	12-Month Median (Q1–Q3)	*p* *
Total number of syncopes	3 (1/5)	0 (0/0.25)	0.00 *
Traumatic events	0 (0/1)	0 (0/0)	0.00 *
Epworth Scale	8 (2 /14)	5 (0/8)	0.00 *
AHI	23.5 (10.9/38.5)	5 (1.9/9.3)	0.00 *
ODI3	22.9 (9.9/39)	5.2 (2.18/11.5)	0.00 *
T90	6.5 (0.7/16.4)	0.2 (0/4.5)	0.00 *
Average RR (ms)	936 (820.2/1018)	991 (892.5/1112)	0.00 *
SDNN (ms)	100.5 (73/157.3)	95.5 (76.3/144.3)	0.50
SDNN index (ms)	74 (47/111-8)	72 (49.7/120.3)	0.26
RMSSD (ms)	68 (39/151.8)	71 (41/178)	0.04 *
NN50	1942 (473.3/6575)	1991 (757/5798)	0.76
%NN50	8.55 (2.28/29.6)	10.5 (3.2/31.1)	0.23
SDANN (ms)	59.5 (39.5/104)	57.5 (41.3/99.5)	0.76
Average total power (ms^2^)	21,538 (12,667/34,247)	22,107 (13,394/36,478)	0.86
Average VLF power (ms^2^)	10,211.5 (2970.3/19,477.5)	9475.5 (1924.5/18,137.8)	0.37
Average LF power (ms^2^)	6848 (3478/11,344)	6998 (3978/12,849)	0.73
Average HF power (ms^2^)	3170 (2132/4920)	3399 (2089/5173)	0.29
LF/HF	2.17 (1.25/3.4)	1.74 (0.96/3.53)	0.26
Triangular index HRV	16 (12/19.5)	15 (10/20)	0.84
Visual analogue scale	50 (40/70)	70 (50/70)	0.00 *
SF36-Physical functioning	65 (35/85)	60 (25/82.5)	0.18
SF36-Role physical	59.5 (75/31)	63 (38/88)	0.49
SF36-Bodily pain	51 (31/74)	51 (22/84)	0.99
SF36-General health	45 (30/60)	45 (35/55)	0.62
SF36-Vitality	44 (16/63)	50 (31/69)	0.02 *
SF36-Social functioning	63 (50/100)	75 (50/100)	0.31
SF36-Role emotional	75 (50/100)	83 (50/100)	0.50
SF36-Mental health	55 (47.5/72.50)	60 (45/70)	0.86

Abbreviations: AHI—apnea-hypopnea index; ODI3: desaturation index >3%, T90—desaturation time below 90%; ms—milliseconds; HRV—heart rate variability; RR—RR intervals; SDNN—standard deviation of RRs; SDANN—standard deviation of the averages of RR intervals across all segments per minute of the entire analysis interval; RMSSD—square root of the mean value of the sum of the squared differences of all successive RR intervals; NN50—number of consecutive intervals varying by more than 50 ms; HF—high-frequency range power; VLF—very low-frequency range power; LF—low-frequency range power. * = *p* < 0.05.

## Data Availability

The data presented in this study are available upon request from the corresponding authors.
